# A Flexible Temperature Sensor Array with Polyaniline/Graphene–Polyvinyl Butyral Thin Film

**DOI:** 10.3390/s19194105

**Published:** 2019-09-23

**Authors:** Jin Pan, Shiyu Liu, Hongzhou Zhang, Jiangang Lu

**Affiliations:** 1 National Engineering Lab for TFT-LCD Materials and Technologies, Department of Electronic Engineering, Shanghai Jiao Tong University, Shanghai 200240, China; 2 Shenzhen Goodix Technology Co. Ltd., Shenzhen 518000, China

**Keywords:** temperature sensor array, temperature coefficient of resistivity (TCR), polyaniline/graphene (GPANI), polyvinyl butyral (PVB) thin film

## Abstract

Thermal-resistance temperature sensors generally employ temperature-sensitive materials as active layers, which are always deposited on a flexible substrate to improve flexibility. Such a temperature sensor is usually integrated in wearable devices with other sensors, such as pressure sensors and stretchable sensors. In prior works, the temperature and pressure sensors are usually located in different layers in a multifunction sensor, which results in a complicated fabrication process, as well as a large thickness of devices. Meanwhile, many temperature sensors are based on large areas of non-transparent materials, leading to difficulties in integrating display applications. In this paper, we demonstrate a flexible temperature sensor based on polyaniline/graphene (GPANI)–polyvinyl butyral (PVB) thin film and indium tin oxides (ITO)- polyethylene terephthalate (PET) substrates. The GPANI particles embedded in PVB film not only contribute to temperature detection, but also response to external pressures, due to weak deformations. In addition, the thin composite film (2.7 μm) highly improved the transparency. By optimizing the device structure, the sensor integrates temperature and pressure detection into one single layer, which shows a wide temperature range of 25–80 °C, a pressure range of 0–30 kPa, and a high transparency (>80%). The temperature sensor offers great potential for applications in emerging wearable devices and electronic skins.

## 1. Introduction

Various studies on wearable devices and electronic skins based on pressure sensors and stretchable sensors have been carried out. Temperature detection is actually another important factor to be integrated in wearable devices, in order to monitor the body temperature and response to the ambient temperature, similar to real skins. There are many kinds of temperature sensors, such as thermal-couple sensors [1], infrared temperature sensors [2], optic fiber temperature sensors [3], thermal-resistance temperature sensors [4,5], and some sensors based on thermal response field-effect transistors [6,7]. The infrared and optic fiber sensors are usually for non-contact temperature detection. The thermal-couple type, thermal-resistance type, and transistor-based type are actually the appropriate candidates for electronic skins, because they can detect contact temperature. A thermal-resistance temperature sensor is generally based on temperature-sensitive materials, such as metals [8], semiconductors [9], and polymers [10,11,12,13]. The metal and semiconductor are usually deposited on a flexible substrate to improve flexibility. Compared with metal and semiconductors, a polymer with conductive temperature-sensitive materials shows greater flexibility for a low Young’s Modulus. Such a temperature sensor based on polymers can usually detect temperature in two mechanisms. One is the deformation of the active layer [10,11], and another is the conductivity change of temperature sensitive materials [12,13]. Both mechanisms always work at the same time.

In prior creations, the pressure array and temperature array are fabricated separately in different layers and then packaged into a system, which results in a complicated fabrication process as well as a larger thickness of devices. Meanwhile, conventional temperature sensors are usually based on large areas of non-transparent conductive materials, such as Pt, graphene, and poly(3,4-ethylenedioxythiophene):poly(styrenesulfonate) (PEDOT:PSS), leading to poor transparency. To get a facile fabrication and high transparency, in this paper, we proposed a temperature sensor based on polyaniline/graphene (GPANI)–polyvinyl butyral (PVB) composite film, and optimized the sensor’s structure. The GPANI shows high conductivity and is sensitive to ambient temperatures, making it a suitable material for the temperature sensor. The sensor integrates the temperature layer and pressure layer into one single layer, and the thin composite film with PVB and GPANI particles shows a high transparency (>80%). The sensor shows a wide temperature range of 25–80 °C, which can detect body temperature. Meanwhile, the sensor can detect both pressure and temperature under low pressure, and only temperature under high pressure, which exhibits the potential to be used in wearable devices in the future.

## 2. Materials and Methods

**Figure 1:**
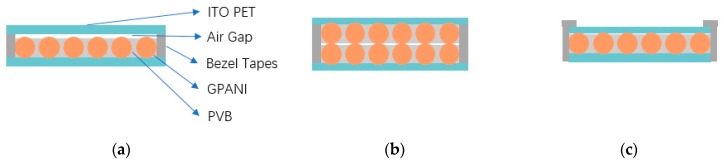
Different structures of sensors. (**a**) Top and bottom substrates directly bonded with bezel tapes or spaces in between, with only one polyaniline/graphene (GPANI)–polyvinyl butyral (PVB) film between the top and bottom electrodes; (**b**) two GPANI–PVB films between the top and bottom electrodes; (**c**) top and bottom substrates fixed by bezel tapes at the outside periphery of PET substrates, with one GPANI–PVB film.

Based on our previous work [14], we optimized the sensor’s structure. In previous design, a GPANI–PVB film is sandwiched between two ITO-PET films. Because the bezel tapes contacting the top and bottom substrates have a larger thickness than the GPANI–PVB composite film, there is a thin air gap between the GPANI–PVB film and the top PET substrate (Figure 1a). Such a thin air gap usually shows expansion and shrinkage when pressure changes, which results in a change of contact areas between the active layer and ITO electrodes and improves the sensitivity of the device. However, the air gap shows adverse effects when the sensor is used for detecting temperatures. Because the air shows low thermal conductivity, it is not good for heat conduction but is suitable for heat preservation.

To eliminate the air gap between the top ITO-PET substrate and the GPANI–PET composite film, we designed two structures. In the first structure, two GPANI–PET films are fabricated between top and bottom electrodes (Figure 1b). Although the thickness of the two films is the same as the bonding tapes, such a structure shows a large resistance (>20 MΩ), due to the difficulties in self-alignment of GPANI particles on the top and bottom films. In the second structure, the bezel tapes are at the outside periphery of the PET substrates (Figure 1c), which showed a great conductivity and apparently reduced the thickness of the air gap. Therefore, the structure in Figure 1c, with a single active layer and outside bonding, is the candidate for our temperature sensor.

Polyaniline (PANI) is a temperature sensitive material and it is easy to aggregate [15]. However, GPANI can tackle the aggregation of polyaniline and retain high conductivity and dispersity. To get a homogenous solution with polyaniline uniformly dispersed in the solution, we used GPANI as the temperature sensitive material. Generally, GPANI can be synthesized with graphene oxide, reduced graphene oxide, or graphene. Among the three kinds of graphene, the GPANI based on graphene oxide shows the highest dispersity and lowest conductivity. The GPANI based on graphene shows the lowest dispersity and highest conductivity. The dispersity and conductivity of the GPANI based on reduced graphene oxide are between those of other two. Since we need a film with GPANI particles uniformly dispersed within it, the GPANI based on graphene oxide was used. The oxidation state of PANI is emeraldine, due to its high stability.

**Figure 2:**
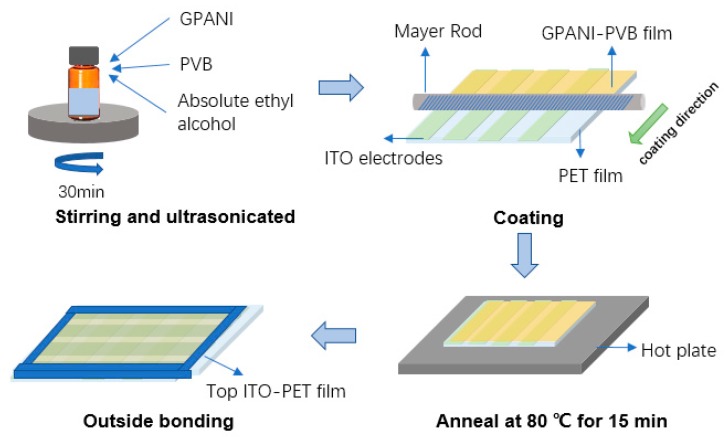
The fabrication process of the sensor.

The fabrication process is shown in Figure 2. First of all, the GPANI (HQNANO-GR-030, Tanfeng Tech. Inc., Suzhou, China), PVB (30153960, Sinopharm Chemical Reagent Co., Ltd., Shanghai, China), and absolute ethyl alcohol (80059490, Sinopharm Chemical Reagent Co., Ltd., Shanghai, China) were stirred for 4 h and ultrasonicated for 30 min to make a homogenous solution. The weight ratio of the GPANI, PVB, and absolute ethyl alcohol was 0.1:5:100. The concentration of GPANI showed no influence on the sensitivity of the temperature sensor, since the temperature only affected the mobility of the GPANI particles. The resistance of the GPANI particles can be expressed as Equation (1), where *R_GPANI_* represents the resistance of the GPANI particle, *ρ* is the resistivity of the GPANI, *σ* is the conductivity of the GPANI, *μ* is the mobility of the GPANI, *n* is the number of carriers, *L* is the length of the GPANI particle, and *S* is the cross-section area of the particle. The resistance of the sensor can be defined as Equation (2), where *D* represents the concentration of the GPANI particles. The sensitivity of the sensor can be expressed as Equation (3), where *S_sensor_* is the sensitivity of the sensor, *R_s_* is the resistance of the sensor, *R_s_*_0_ is the initial resistance of the sensor, and *μ*_0_ represents the initial mobility of the GPANI. According to Equation (3), the sensitivity *S_sensor_* only depends on the mobility (*μ*) and the number of carriers (*n*). Both *μ* and *n* are affected by temperatures. Therefore, the sensor can be used for temperature detection, and the sensitivity of the sensor is not related to the concentration of GPANI on the device.

(1) $$R_{GPANI}=\rho \frac{L}{S}=\frac{L}{\sigma S}=\frac{L}{\mu neS},$$

(2) $$Rs=D\times R_{GPANI}=\frac{DL}{\mu neS},$$

(3) $$S_{sensor}=\frac{R_{s}-R_{s0}}{R_{s0}}=\frac{\frac{DL}{\mu neS}-\frac{DL}{\mu_{0}n_{0}eS}}{\frac{DL}{\mu_{0}n_{0}eS}}=\frac{\mu_{0}n_{0}-\mu n}{\mu n}.$$

**Figure 3:**
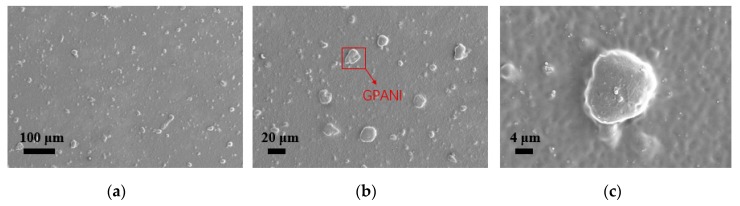
The scanning electron microscopy (SEM) image of the distribution of GPANI in PVB films on the scale of (**a**) 100 μm; (**b**) 20 μm; (**c**) 4 μm.

When the ratio of GPANI increased, the transparency decreased. When the ratio of GPANI decreased, the resistance of the sensor increased. To get a high transparency and low resistance, we used 0.1 wt % GPANI. Meanwhile, as a large thickness of PVB will decrease the transparency, the PVB of 5 wt % was used. The ethyl alcohol was served as a third solvent, because GPANI particles can be well dispersed in alcohol. Figure 3 is the scanning electron microscopy (SEM) (WT2ZSEM01, Zeiss Co., Ltd., Jena, Germany) image, and the GPANI particles are uniformly distributed in the PVB film.

The mixture was then uniformly coated on an ITO-PET substrate with a Mayer rod (Bar No. 30, R.D.SPECIALTIES. Inc., New York, NY, USA) to make a wet GPANI–PVB film. The PET film had a thickness of 0.13 mm, an area of 75 mm × 75 mm, and 10 strip ITO electrodes with a width of 4 mm. The electrodes were parallelly aligned on the PET film. with an interval of 1 mm. The wet film of the composite was then annealed at 80 °C for 15 min to eliminate the alcohol, and the thickness of the dry composite film was 2.7 μm. A second ITO-PET was then coated directly on the film without any spacers, and the ITO electrodes on the top PET film were aligned perpendicularly to the electrodes on the bottom PET film. The top and bottom substrates were finally fixed by bezel tapes at the outside periphery of the PETs. Then, a temperature sensor with GPANI–PVB composite film was fabricated.

## 3. Results and Discussion

Compared with polyaniline with poor conductivity, the GPANI shows a high conductivity and good dispersity. The conductivity of GPANI is sensitive to ambient temperature, which is caused by two competing effects. On the one hand, there are more and more additional carriers when the temperature increases. One the other hand, the scattering of the lattice gets more and more intense as the temperature increases, which decreases the mobility of carriers. Generally, only one of these two effects dominates the conductivity of GPANI particles. Under low temperature, the conductivity is dominated by the first effect. The mobility, as well as the numbers of carriers, increases dramatically, due to more and more absorbed energy. As the temperature increases to a threshold temperature, the mobility decreases dramatically due to lattice scattering, and the second effect dominates. The GPANI particles were embedded in and penetrated through the transparent PVB films, leading to a top-down conduction of sensors. Since GPANI particles have slight deformations under low pressure, the resistance is subject to both the pressure and temperature. Therefore, the sensor with one single active layer can be used to detect pressure as well as temperature under 30 kPa. When the pressure is larger than 30 kPa, there is no further deformation in GPANI particles, and the pressure has almost no effect in the resistance of sensors. The resistance of the sensor is only affected by temperatures, which leads to a single temperature measurement.

The measurement was performed by applying certain pressure on the sensor with a pressure meter (ZQ-20B-1, Zhiqu Co., Dongguan, China). We also used a temperature controller (HCS302, Instec Co., Boulder, CO, USA) to apply different temperatures. The resistance was measured by a multimeter (Vx890C+, Victory Co., Shenzhen, China) at a humidity of 75%. 

**Figure 4:**
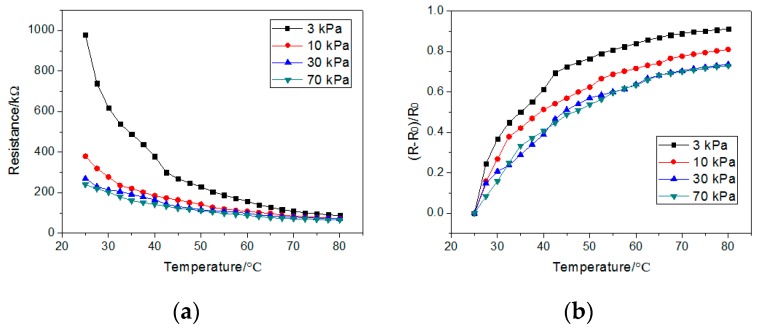
(**a**) The resistance and (**b**) the normalized resistance change, (*R* − *R*_0_)/*R*_0_, under different pressures, at 25–80 °C on temperature sensors and with humidity at 75% in (5,5).

As Figure 4a shows, the resistance decreased in the temperature range of 25–80 °C at 75% humidity, since the absorbed energy accelerated the carriers and increased the number of carriers. We did not measure the performance of the temperature sensor above 80 °C, because the PET substrate shows irreversible deformation above that temperature. As the GPANI particles show weak deformations under 30 kPa, the resistance varies from 0–30 kPa at the same temperature, which leads to a detection of both pressure and temperature. When the pressure is higher than 30 kPa, there are no more deformations in the GAPNI particles. The resistance stays nearly stable, and only changes with temperature, which allows for a single detection of temperature. The normalized resistance change (*R* − *R*_0_)/*R*_0_ is shown in Figure 4b.

**Figure 5:**
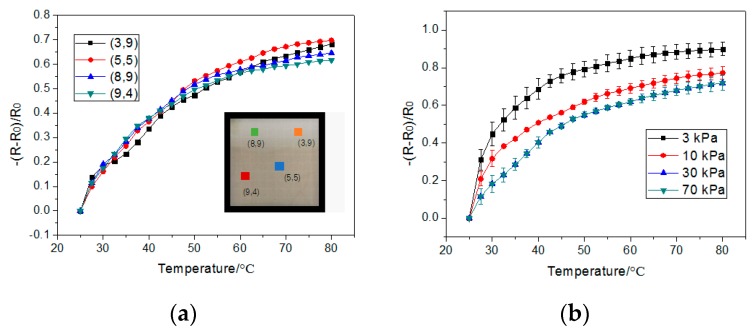
(**a**) The resistance change in (5,5), (3,9), (8,9), and (9,4) at 25–80 °C, 70 kPa, and 75% humidity; (**b**) the average resistance changes under 3, 10, 30, and 70 kPa at 25–80 °C, with a humidity of 75%.

The sensor allows multi-points measurements, since the normalized resistance change (*R* − *R*_0_)/*R*_0_ is consistent on different points in the sensor array, as shown in Figure 5a. Figure 5b shows the average resistance change in (5,5), (3,9), (8,9), and (9,4) under different pressures and different temperatures, and the small error bar represents the variance of the average resistance changes, which shows a high consistency on different points of the sensor array. 

**Figure 6:**
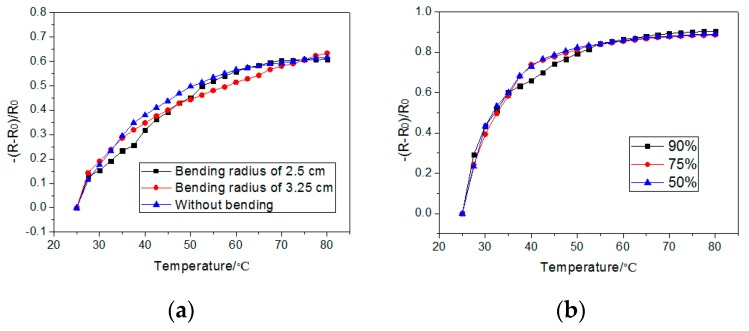
The response of the sensor at 25–80 °C in (5,5) (**a**) with a different bending radius at 70 kPa, and (**b**) with different humidity at 3 kPa.

The response of the sensor under bending is shown in Figure 6a. The sensor showed a similar response when the bending radius was 2.5 and 3.25 cm. The humidity shows little influence on the sensor, for the sensor has an encapsulation, with bezel tapes bonding the top and bottom PET films at the outside periphery; in addition, the PET is waterproof, as shown in Figure 6b.

As the resistance is affected by both temperature and pressure change under 30 kPa, we proposed a method to get the target temperature and pressure. First, a model showing the relationship between temperature, pressure, and resistance can be obtained by fitting the measured data with multiple linear regression function in simulation software Matlab. The model shows a quadratic equation of binary variables temperature (*T*) and pressure (*P*), as Equation (4) shows. Then the measured resistances can be substituted into Equation (1) to get the corresponding *T* and *P*. For example, in a heating process with constant pressure, since the conduction of temperature takes a certain amount of time, we can get *N* resistances [*R*_0_…, *R_i_*…, *R_N_*], where *Ri* represents the resistance at an intermediate temperature. Then, we can substitute these *N* resistances into Equation (4) to get the target pressure and temperature.

(4) $$(R_{0}-R)/R_{0}=-2.9983\times 10^{-4}T^{2}+1.1206\times 10^{-4}P^{2}-2.8391\times 10^{-6}TP+0.0429T-0.0115P-0.6011.$$

The temperature coefficient of resistivity (TCR) α is defined in Equation (5). Our temperature sensor shows a negative TCR of −1.2% °C^−1^ at 25–80 °C, which is higher than the temperature sensors with Pt and PEDOT:PSS–carbon nanotube (CNT). The TCR of the sensor is also larger than that of the sensor based on polyaniline nanofibers [4]. Moreover, our temperature sensor has a much high transparency, as shown in Table 1.

(5) $$\alpha =\frac{1}{R(T_{0})}\times \frac{R(T)-R(T_{0})}{T-T_{0}}.$$

**Figure 7:**
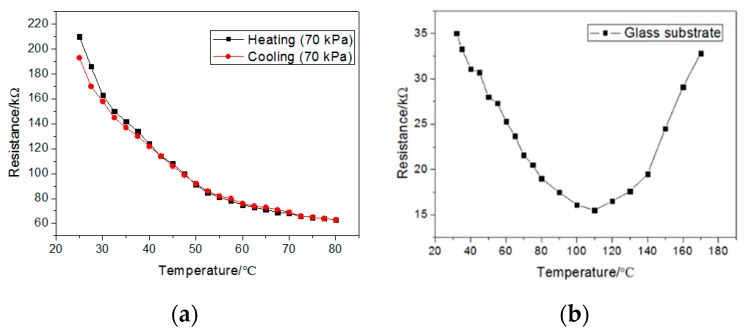
(**a**) The resistance in a cycle of heating and cooling in (5,5) at 25–80 °C. (**b**) The resistance of the temperature sensor with ITO–glass substrates in (5,5) at 30–170 °C.

The sensor shows a little hysteresis in a cycle of heating and cooling from 25 to 80 °C, as shown in Figure 7a. Compared with many previous works, the temperature sensor shows a high transparency (>80%). The high transparency contributes to both the small diameters of GPANI particles (10–20 μm) and the transparent thin PVB films (2.7 μm). As a result, the sensor shows potential for being integrated with other interactive applications, such as displays.

To confirm the mechanism of the temperature sensor based on GPANI-PET films, we replaced ITO–PET substrates with ITO–glass substrates, which can sustain temperatures higher than 100 °C. The performance of the sensor is showed in Figure 7b. The resistance decreased at low temperatures, and began to increase until the temperature became higher than 120 °C, which implies that the absorbed energy increased the conductivity of carriers below 120 °C, and then lattice scattering began to dominate and decreased the conductivity of GPANI particles above 120 °C. Therefore, the GPANI–PVB composite film can detect a larger temperature range when using electrodes with higher heat resistance.

## 4. Conclusions

In summary, we proposed a temperature sensor based on GPANI–PVB composite film. The sensor shows a negative temperature coefficient of resistivity (−1.2%·°C^−1^) in the temperature range of 25–80 °C. The resistance of the GPANI–PVB film decreased below 120 °C and increased when the temperature was higher than the threshold temperature, which enables the detection of a broader temperature range by using substrates with higher heat resistance. The temperature sensor allowed detection of both pressure and temperature under low pressure (<30 kPa), and integrated the pressure and temperature sensors into one single layer. Meanwhile, the sensor can only detect temperature under high pressure (≥30 kPa), because the GPANI particle shows no more deformation under such pressure. Moreover, high transparency (>80%) made the temperature sensor a potential candidate for wearable devices, which can be well integrated with other visual, interactive applications, such as displays.

## Figures and Tables

**Table 1:** Comparation of the sensor in this study and sensors in prior works.

Materials of Active Layer in Sensors	|TCR|(%·°C^−1^)	Transparency
GPANI–PVB	1.2	>80%
Pt [16]	0.385	No
PEDOT:PSS-CNT [12,13]	0.2–0.6	No
Polyaniline [4]	1.0	No
Multiwalled carbon nanotube [17]	0.07	No
